# Duan-Nai-An, A Yeast Probiotic, Improves Intestinal Mucosa Integrity and Immune Function in Weaned Piglets

**DOI:** 10.1038/s41598-020-61279-6

**Published:** 2020-03-12

**Authors:** Yingpai Zhaxi, Xiaoqin Meng, Wenhui Wang, Ling Wang, Zhuolin He, Xuejing Zhang, Wanxia Pu

**Affiliations:** 1 Key Laboratory of New Animal Drug Project, Gansu Province and Key Laboratory of Veterinary Pharmaceutics Discovery, Ministry of Agriculture and Rural Affairs, Lanzhou Institute of Husbandry and Pharmaceutical Sciences of CAAS, Lanzhou, 730050 China; 2 College of Life Sciences and Engineering, Northwest Minzu University, Lanzhou, 730030 China; 3 Lanzhou Center for Animal Disease Control and Prevention, Lanzhou, 730050 China; 4 College of Veterinary Medicine, Gansu Agricultural University, Lanzhou, 730070 China

**Keywords:** Diarrhoea, Drug development, Duodenum, Ileum, Jejunum

## Abstract

Post-weaning diarrhea commonly occurs in piglets and results in significant economic loss to swine producers. Non-antibiotic measures for managing post-weaning diarrhea are critically needed. Duan-Nai-An, a probiotic produced from the yeast fermentation of egg whites, was previously shown to optimize intestinal flora and reduce the incidence of clinical diarrhea in weaning piglets. To study the effects of Duan-Nai-An on mucosal integrity and immunity in pig intestine, we examined the microstructure and ultrastructure of the intestines of weaned pigs with or without Duan-Nai-An as a feed supplement. The piglets of the Duan-Nai-An-fed group developed intestines with intact columnar epithelia covered by tightly packed microvilli on the apical surface. However, piglets of the control group (no supplement) showed villous atrophy and thinning, microvillus slough, and in the severe cases, damage of intestinal epithelia and exposure of the underlying lamina propria. Moreover, piglets of the Duan-Nai-An-fed group showed apparent plasmocyte hyperplasia, increased lymphoid nodule numbers, well-developed Peyer’s Patchs, and apparent germinal centers. The lymphoid tissues of the control group were far less developed, showing lymph node atrophy, lymphocyte reduction, degeneration, and necrosis. These results indicate that Duan-Nai-An improves the development of the intestinal structures and lymphoid tissues and promotes intestinal health in weaned piglets.

## Introduction

The gastrointestinal (GI) tract is the largest immune organ in the body^1,2^. The epithelial lining of the GI tract is the surface that is in close contact with the external environment. The GI tract also provides the microenvironment for the development and differentiation of the intestinal immune cells. The intestinal epithelial cells play important roles not only in food digestion and nutrient absorption, but also in immune defense and immune tolerance^3^. More than 50% of the body’s lymphoid tissue is distributed in the intestinal mucosa, bearing an important immune function and constituting the body’s first barrier against pathogenic microorganism invasions^4^.

A diverse and large number of microorganisms are found in the GI tract. Gut microbiota play very important roles in the growth, anti-infection, immune regulation, metabolism and intestinal health of the host^5–8^, and most studies mainly focused on evaluating the impact of bacterial flora on animal nutrition^9–11^. Recently, more and more work has been devoted to understanding the microbiota-intestinal mucosa interaction, how the interaction form a mechanical, immunological, and biological barrier against pathogen invasion and endotoxin translocation, and how gut microbiota modulates the development and function of the mucosal immune system^5,12–15^.

Weaning is one of the most stressful events in the swine production process and disturbs the intestinal flora of piglets, often compromising the intestinal structure and mucosal barrier^16^. Because of the disruption, weaning piglets may have decreased intestinal mucosal immunity^17^ and develop symptoms including diarrhea, poor appetite, and slow growth. This results in serious economic loss to the swine industry^18,19^. Therefore improving the intestinal barrier function may provide an effective way to ease the stress of weaning.

*Saccharomyces cerevisiae* fermented egg white, known as active egg white product (AEWP)^20^, was reported to improve macrophage functions in mice^20^ and to promote activation of neutrophilic functions in piglets^21^ and calves^22^ by oral administration, therefore enhancing host resistance to infections. In order to improve the growth performance of early-weaned piglets, we tamed a *S. cerevisiae* strain S288c to grow well in egg white, and developed a yeast probiotic by fermenting egg white, which was named Duan-Nai-An. Thus, Duan-Nai-An was a combination of live yeast and active egg white. Our previous studies showed that tamed *Saccharomyces cerevisiae* fermented egg white (Duan-Nai-An) was effective to maintain a stable bacterial community in gut microbiota and to reduce the incidence of diarrhea for early-weaned piglets^23^. To further understand how Duan-Nai-An improves intestinal health in piglets, in this study, Duan-Nai-An and another probiotic that was prepared as original *S. cerevisiae* S288c fermented malt were examined as feed supplements to the diet of weaning piglets. The effect of Duan-Nai-An on the intestinal structure and mucosal immunity were evaluated at the age of 23 to 42 days during the probiotic feeding and after weaning by light microscopy and electron microscopy.

## Results

### Clinical sign and changes in gross anatomy

**Figure 1:**
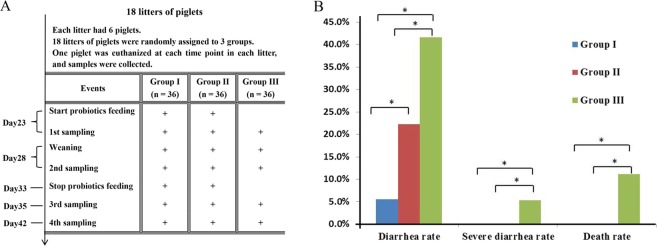
Experimental design of the study and clinical observations of the piglets. (**A**) Group assignment and sampling scheme. Group A was supplemented with probiotic Duan-Nai-An, Group B was supplemented with *Saccharomyces cerevisiae* fermented malt, and Group C was used as a base diet control without any probiotics; (**B**) Diarrhea rate, severe diarrhea rate, and death rate among the group. Significant differences (P < 0.05) between the groups were indicated by stars.

Eighteen litters of crossbred piglets (Duroc × Yorkshire × Landrace) were used for this study. The experimental design and sampling schemes were depicted in Fig. 1A.

During the entire process of the experiment, piglets of the Duan-Nai-An-treated group had the least clinical signs of diarrhea compared to other groups (Fig. 1B). Diarrhea rate, severe diarrhea rate, and death rate of Group I and Group II were significantly lower (P < 0.05) than those of Group III. Furthermore, Group I had lower diarrhea rate (P < 0.05) than Group II (Fig. 1B). The average onset ages of diarrhea were 36 and 34 days, respectively, for Group I and Group II, while it was 30 days in Group III. The average duration of diarrhea of Group I and Group II was 6 and 7 days respectively, while the duration of Group III as 9 days (P < 0.05).

**Figure 2:**
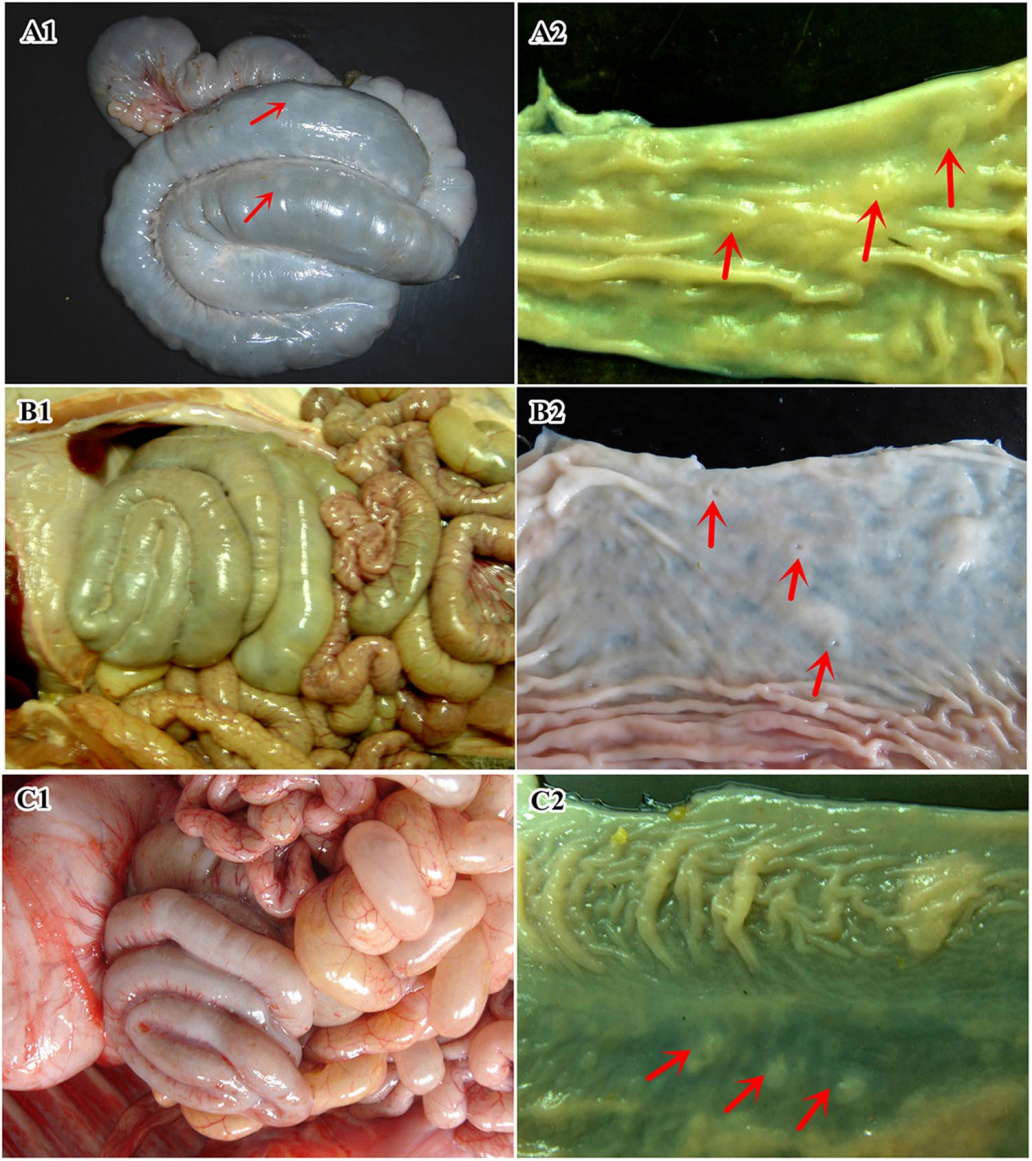
Representative anatomic features of colon in 42-day old piglets. In all panels, red arrows indicate Peyer’s Patchs (PPs)**. (A1**) The serosal side of colon of a Duan-Nai-An-treated piglet. The colon was normal and stout, with greyish-white PPs. (**A2**) The mucosal surface of colon from a Duan-Nai-An-treated piglet. The colon was thick and stout. There were a large number of crateriform yellowish-white PPs. (**B1)** The serosal side of colon from a yeast-treated piglet. The colon appeared normal. (**B2**) The mucosal side of colon from a yeast-treated piglet. The crateriform PPs were visible. (**C1**) The serosal side of colon from a control piglet. The colon was severely under-developed, dilated, and congested. (**C2**) The mucosal side of colon from a control piglet. The crateriform PPs was visible.

For the changes in gross anatomy, there was not much difference among the three groups at 23 and 28 days of age. While at 35 days of age, Duan-Nai-An-treated piglets showed normal and healthier GI tract with Peyer’s Patches (PPs) on the colonic surface compared to the other two groups. The piglets in the control group showed smaller intestine both in diameter and length and thinner intestinal wall, as well as intestinal congestion. At 42 days of age, the piglets of Duan-Nai-An-treated group developed thick, normal, and stout colons, with well-developed grey PPs on the surface (Fig. 2A1). On the mucosal surface, there were many scattered crateriform yellowish-white PPs (Fig. 2A2). The colon of piglets in the yeast-treated group at 42-day age appeared normal (Fig. 2B1). On the mucosal surface, the crateriform PPs were visible (Fig. 2B2). In contrast, piglets of the control groups at 42-day age showed poorly-developed colons with few PPs distribution and partly dilated small intestine with gas (Fig. 2C1,C2). The dead piglets in the control group demonstrated severe developmental disorder in colon, dilated small intestine, and severe intestinal congestion in the whole GI tract (data not shown).Together, the results showed that Duan-Nai-An significantly decreased the incidence of diarrhea and alleviated the clinical sign of diarrhea, and at the meantime improved intestinal health in piglets.

### Effect of Duan-Nai-An on the histology of intestinal mucosa

#### Duodenum

At 23 and 28 days of age, the duodenum from piglets of the different treatment groups showed intact villi, normal lamina propria, and abundant goblet cells in the intestinal glands. However, some differences were observed, in which the duodenum from Duan-Nai-An-treated piglets demonstrated thicker and stronger villi, more dense lymph cells in lamina propria, and more intact microvilli than pigs in other groups. At 35 days of age, Duan-Nai-An-treated piglets showed further intestinal development indicated by thicker villi and more cells in the lamina propria. Duodenum from the yeast-treated group (Group II) showed no changes compared to those of 28-day old piglets in the same treatment group. However, duodenum from the control piglets (Group III) had thinner villi, reduced number of cells in the lamina propria, and partial loss of microvilli.

**Figure 3:**
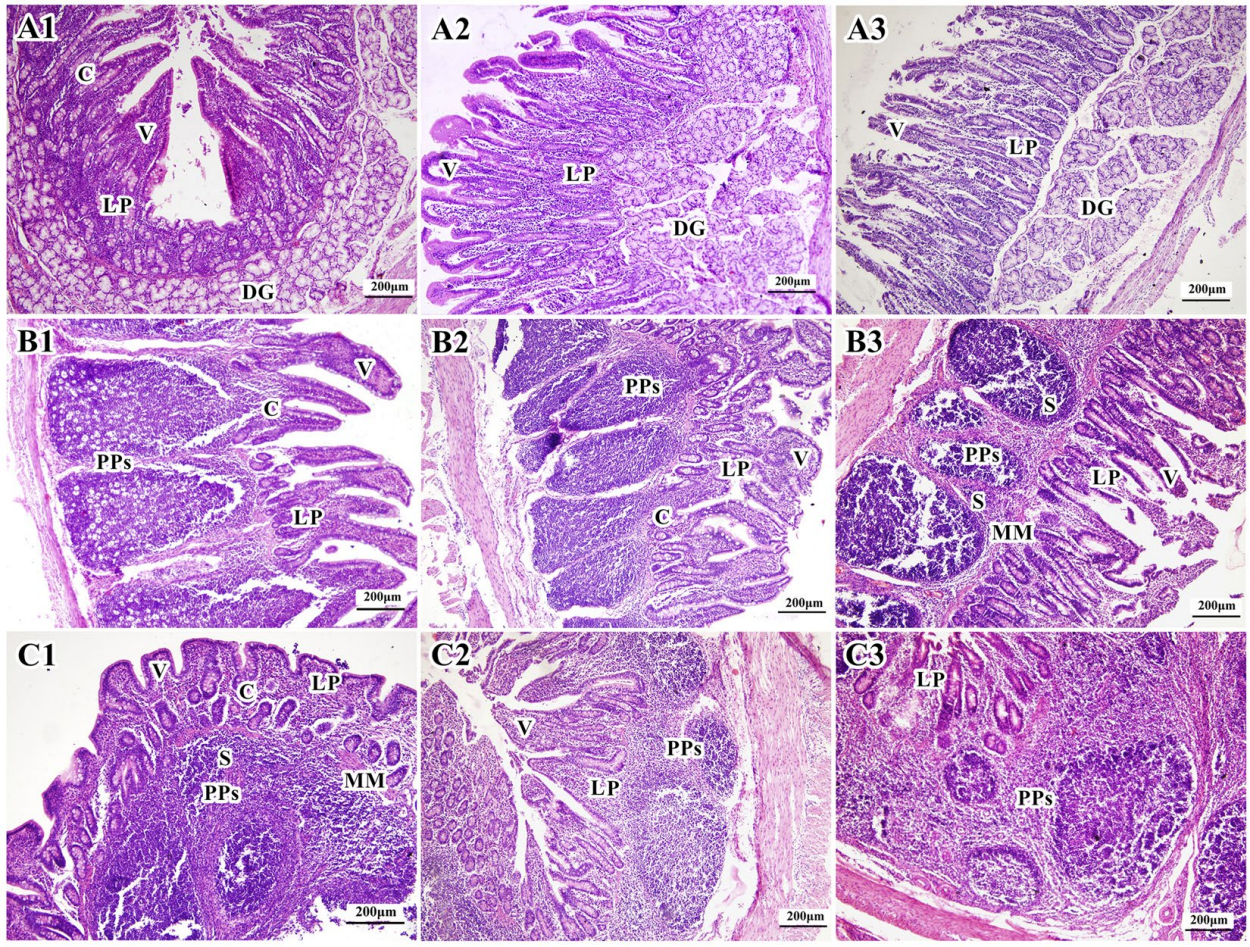
Histological structures of small intestinal mucosa in 42-day old piglets (stained with hematoxylin-eosin). In all panels, the letters indicate the following structures: C, Crypts; DG, duodenal gland; LP, Lamina propria; MM, muscularis mucosae; PPs, Peyer’s Patches; S, submucosa; and V, villi. (**A1**) Duodenum of a Duan-Nai-An-treated piglet. The duodenal villi were thick and strong. There were a great number of lymphocytes in the lamina propria. (**A2**) Duodenum of a yeast-treated piglet. There were a great number of lymphocyte cells in the lamina propria. (**A3**) Duodenum of a control piglet. The duodenal villi were thin and atrophied. Part of the epithelial cells sloughed off. (**B1**) Jejunum of a Duan-Nai-An-treated piglet. There were well-developed PPs observed in the jejunal submucosa and a great number of cells in the lamina propria. The jejunal villi were intact. (**B2**) Jejunum of a yeast-treated piglet. The PPs in the submucosa of jejunum were well-developed. There were a number of lymphocytes in the lamina propria. (**B3**) Jejunum of a control piglet. The PPs in the submucosa of jejunum were visible. Jejunal villi were atrophied and epithelial cells sloughed off. (**C1**) Ileum of a Duan-Nai-An-treated piglet. The PPs in the submucosa were very well-developed. There were a large number of lymphocytes in the lamina propria. (**C2**) Ileum of a yeast-treated piglet. The PPs in the submucosa were well-developed. There were a large number of lymphocytes in the lamina propria. (**C3**) Ileum of a control piglet. The PPs in the submucosa were visible. Ileal villi were atrophied and epithelial cells sloughed off.

At 42 days of age, the duodenum of the Duan-Nai-An-treated group had thick and healthy villi, numerous lymph cells in the lamina propria, and intact microvilli (Fig. 3A1). No obvious changes were observed in the duodenum of the yeast-group compared to days 35 (Fig. 3A2). However, in the duodenum of the control group, the intestinal wall became thin with villous atrophy (thin and shortened villi); the number of lymph cells in the lamina propria decreased significantly; and patches of microvilli fell off (Fig. 3A3). As shown in Table 1, it is worth mentioning that the duodenal villi in Duan-Nai-An-treated piglets were higher and wider than that in control group (P < 0.05), Crypts were deeper in control-group piglets than those in Duan-Nai-An-treated group and yeast group, but there was no significant difference between three groups (P > 0.05). Villous height/crypt depth (V/C) ratio was larger in Duan-Nai-An-treated group than that in yeast and control group (P < 0.05).

**Table 1:** Histological data of intestinal villi and crypts in 42-day-old piglets.

		Groups			*P* value		
		I (n = 6) (M ± SD)	II (n = 6) (M ± SD)	III (n = 6) (M ± SD)	I - I	I - III	I - III
Duodenum	Height	497.30 ± 38.38^a^	388.09 ± 53.04^ab^	346.93 ± 41.89^b^	0.051	0.394	0.015
	Width	179.70 ± 16.96^a^	149.88 ± 16.33^ab^	124.59 ± 16.17^b^	0.121	0.176	0.016
	Depth	204.75 ± 13.33^a^	232.56 ± 32.06^a^	259.15 ± 20.95^a^	0.280	0.299	0.059
	V /C	2.46 ± 0.48^a^	1.72 ± 0.03^b^	1.36 ± 0.10^b^	0.031	0.274	0.007
Jejunum	Height	564.08 ± 23.76^a^	522.95 ± 16.13^b^	508.84 ± 16.15^b^	0.001	0.103	0.001
	Width	298.23 ± 27.42^a^	174.75 ± 13.24^b^	171.99 ± 15.80^b^	0.001	0.827	0.001
	Depth	237.84 ± 13.82^a^	247.65 ± 20.20^a^	249.20 ± 24.76^a^	0.524	0.918	0.463
	V /C	2.38 ± 0.15^a^	2.12 ± 0.06^ab^	2.034 ± 0.05^b^	0.073	0.524	0.029
Ileum	Height	330.07 ± 21.50^a^	288.99 ± 32.62^a^	174.15 ± 29.47^b^	0.196	0.007	0.001
	Width	156.46 ± 45.68^a^	135.11 ± 26.22^a^	136.91 ± 19.35^a^	0.534	0.958	0.568
	Depth	183.55 ± 13.05^a^	220.62 ± 18.40^a^	207.94 ± 29.49^a^	0.134	0.576	0.229
	V/C	1.80 ± 0.06^a^	1.31 ± 0.119^b^	0.86 ± 0.19^c^	0.010	0.014	0.001

^*^V/C = Height of villi /Depth of crypt ratio. Statistical analysis was only conducted in the same row between groups, with different letters indicating significant difference (P < 0.05), and the same letter indicating insignificant difference (P > 0.05).

#### Jejunum

At 23 and 28 days of age, the submucosa of jejunum of all three treatment groups showed PPs and lymphocyte hyperplasia that led to lymphocyte influx into the lamina propria; villi appeared thick and dense; and goblet cells developed in the intestinal epithelial cells. At 35 days of age in the Duan-Nai-An-treated piglets, PPs in the jejunal submucosa further developed; lymphocyte hyperplasia became more apparent; and villi became enlarged. In contrast, in piglets of the yeast-treated group and the control group, PPs were not prominent and some even showed atrophy. The number of lymphocytes in lamina propria of the control groups decreased and villi became thinner.

At 42 days of age, in the Duan-Nai-An-treated piglets, the number of lymphocytes in the lamina propria continued to increase; the PPs were well-developed; and the jejunal villi were not damaged (Fig. 3B1). In yeast-treated piglets, the PPs in the submucosa of jejunum were growth well. There were a number of lymphocytes in the lamina propria (Fig. 3B2). However, in the piglets of negative control-treated group, the villi became thin and atrophied with partial loss of microvilli and the number of lymphocytes in lamina propria decreased (Fig. 3B3). Importantly, jejunal villi in piglets of Group I was higher and wider than those in Group II and Group III (P < 0.05). There were no differences in the depth of crypts between Groups (P > 0.05). V/C ratio was higher in Group I than those in Group III (P < 0.05) (Table 1).

#### Ileum

At 23 and 28 days of age, the ileal submucosa of piglets from all groups was rich with PPs; cell density in lamina propria was high; villi were thick and short; and goblet cells were abundant. At 35 days of age in the piglets from both the Duan-Nai-An- and yeast-treated groups, the PPs grew bigger, lymphocyte hyperplasia led to infiltration of lymphocyte through muscularis mucosa into the lamina propria; and villi were short and thick. In contrast, in the ileum of piglets from the control group, the PPs did not show obvious enlargement, no lymphocytes infiltrated into lamina propria, and the ileal villi were thin and long.

At 42 days of age, the PPs in the submucosa were very well-developed and a large number of lymphocytes infiltrated into the lamina propria as a result of proliferation in the Duan-Nai-An-treated piglets (Fig. 3C1). No obvious changes were observed in yeast-treated piglets. The PPs in the submucosa were well-developed and a large number of lymphocytes in the lamina propria were observed (Fig. 3C2). While in the piglets of control group, ileal villi were atrophied and epithelial cells fell off and the PPs in the submucosa were no obvious enlargement (Fig. 3C3). Furthermore, the heights of ileal villi were larger in piglets of Group I and Group II than those in Group III (P < 0.05), but there were no significant differences between Group I and Group II (I and II; P > 0.05). There was no significant difference in the depth of crypts between the Groups (P > 0.05). The V/C ratio showed a general declining trend from Group I, Group II to Group III, and there were significant differences between the groups (P < 0.05) (Table 1).

#### Cecum and colon

Cecum and colon are part of the large intestine and their main functions are to absorb water and inorganic salts and to decompose cellulose by fermentation. In addition, they secrete mucus to lubricate the intestinal tract. Unlike the small intestine, cecum and colon do not have mucosal folds and villi, and therefore their mucosal surface is smooth. The mucosa of cecum and colon are also rich in glands and goblet cells. The columnar epithelial cells do not have prominent microvilli and thus there are no obvious striated borders. There are isolated lymphoid nodules in the submucosa.

**Figure 4:**
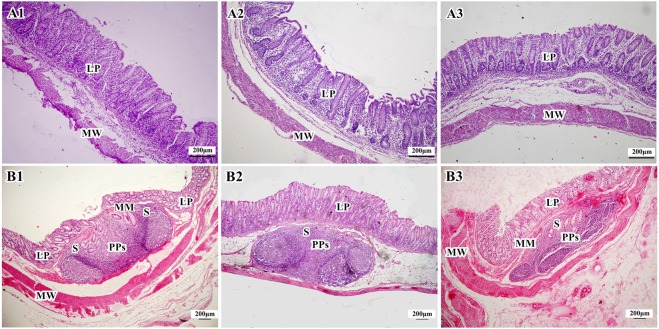
Histological structure of cecal mucosa (A) and PPs in midpiece colon (B) in 42-day old piglets (stained with H&E). In all panels, the structures are indicated by LP, Lamina propria; MM, muscularis mucosae; MW, muscular wall; PPs, Peyer’s Patches; and S, Submucosa. (**A1**–**A3**) Abundant lymphocytes were visible in the lamina propria of ceca from a Duan-Nai-An-treated piglet (**A1**), a yeast-treated piglet (**A2**), and a control piglet (**A3**). (**B1**) In the Duan-Nai-An-treated piglet, PPs were well-developed in the submucosa of midpiece colon and there were abundant lymphocytes in the lamina propria. (**B2**) In the yeast-treated piglet, PPs were well-developed in submucosa of midpiece colon. (**B3**) In the control piglet, PPs were atrophied in the submucosa of midpiece colon and there were congestion in lamina propria.

**Figure 5:**
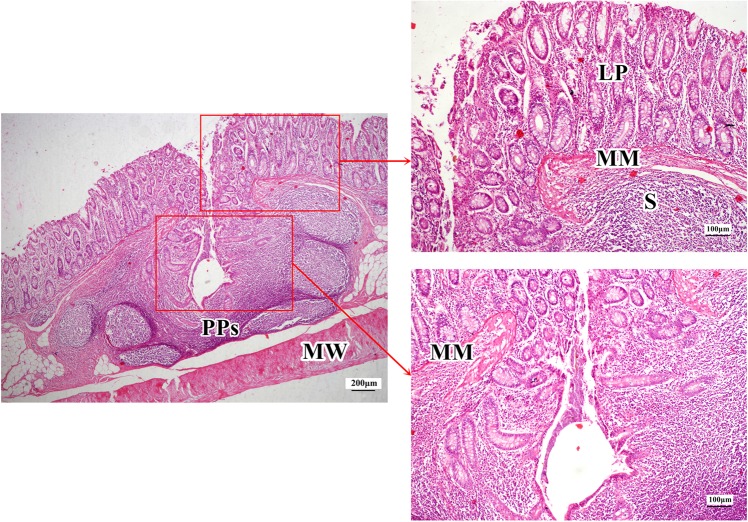
A crateriform PPs in the midpiece colon of a 42-day old Duan-Nai-An-treated piglet (H&E staining). The inner structure of the PPs appeared scrotiform with well-developed lymphoid tissues on the lateral and basal surfaces of the inner wall of the cyst, well-developed lymphoid follicles near the circular muscle layer, and a large number of scattered lymphocytes in the lamina propria. LP indicates Lamina propria; MM indicates muscularis mucosae; MW indicates muscular wall; PPs indicate Peyer’s Patches; and S indicates Submucosa.

There was no obvious difference in the microstructures of cecum among the three groups (Fig. 4A). In the colon of the Duan-Nai-An- and yeast-treated piglets, there were no apparent changes in structures during the experimental period, with clear structural integrity, well-developed glands, a large number of goblet cells in the mucosa, and abundant lymphocytes in lamina propria. Mucosal Peyer’s patches of midpiece colon in 42-day old piglets from the Duan-Nai-An-treated group and the yeast-treated group were well-developed in submucosa (Fig. 4B1,B2). However, in the colon of the piglets of the control group, severe diarrhea led to sloughing of mucosal epithelia cells and the number of lymphocytes in lamina propria were sparse, also the congestion were observed in lamina propria. Peyer’s patches were atrophied in submucosa (Fig. 4B3). There were many crateriform PPs scattered on the surface of colonic mucosa in the 42-day-old weanling piglets of Duan-Nai-An-treated group. As shown in Figure Fig. 5, a crateriform PPs was in the midpiece colon of a 42-day old Duan-Nai-An-treated piglet (H&E). The internal structure of the PPs appeared scrotiform. It had a small opening in the cavity which went directly to the inner surface of intestinal mucosa, making intestinal contents easily enter into cyst of PPs through its opening. The outer surface mucosa of crateriform PPs had no developed lymphoid tissue, but scattered lymphocytes in the lamina propria. The inner cavity of crateriform PPs had developed lymphoid tissue in the whole inner mucosa. There were well-developed lymphoid tissues on the lateral and basal surfaces of the inner of the cyst, with well-developed PPs near the circular muscle and a large number of scattered lymphocytes in the lamina propria.

#### Mesenteric lymph nodes

The swine lymphatic structure is unique in that the lymphoid nodule is aggregated in the center of the lymph node. Undifferentiated germinal centers that contain macrophages and plasma cells are loosely distributed in the periphery of the lymph node. When lymphoid nodules are well developed, the light zone, dark zone, and cap of the germinal center are clearly visible under microscopy, however, these differentiated regions are lost when lymphoid nodules are poorly developed.

**Figure 6:**
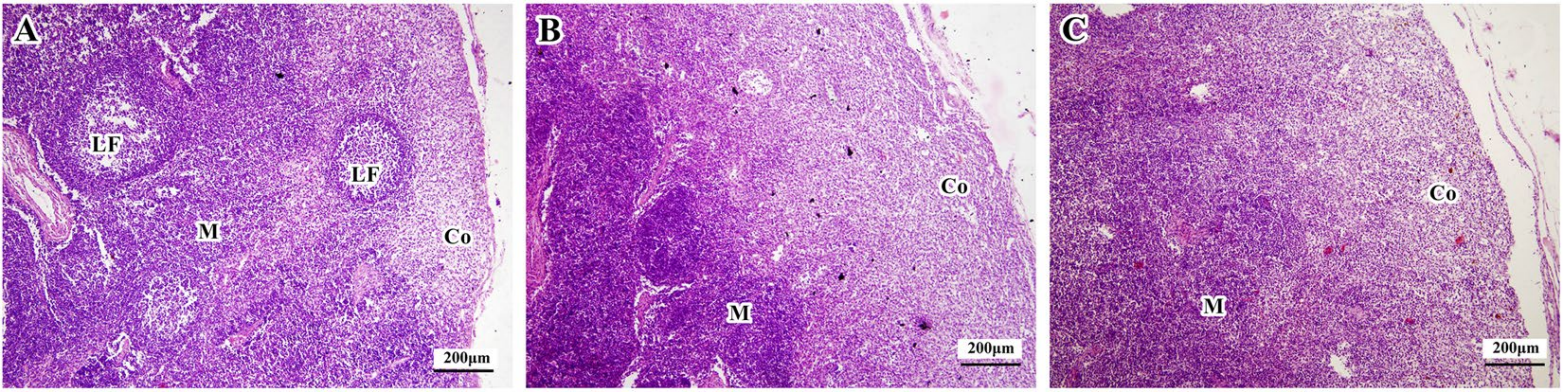
Histological structure of colonic mesenteric lymph nodes in 42-day old piglets (stained with H&E). In all panels, Co indicates cortex; LF indicates lymphoid follicle; M indicates medulla. (**A**) In the Duan-Nai-An-treated piglet, the medulla of lymph nodes or PPs were well-developed with some lymphoid follicles proliferated into the cortex, and germinal centers were obvious. (**B**) In the yeast-treated piglet, the medulla of lymph nodes (or PPs) were well-developed and lymphocytes were dense; (**C**) In the control piglet, the lymph nodes (or PPs) were atrophied and lymphocytes were sparse.

In this study, the lymphoid nodules of the piglets from the Duan-Nai-An and yeast-treated groups were well developed; the light and dark zones of the germinal centers were clearly visible;and some lymphoid nodules extended to the edge of the node under the capsule (Fig. 6A,B). In contrast, piglets of the control group showed poorly developed lymphoid nodule, with no distinguished light and dark zones (Fig. 6C).

### Ultrastructure of the intestinal mucosa observed by Scanning electron microscopy (SEM) and Transmission electron microscopy (TEM)

The changes in the intestinal mucosa of piglets observed under SEM were consistent with those from light microscopy. For this reason, duodenal and ileal mucosa of 42-day old piglets were explained in detail as examples.

#### Duodenum

**Figure 7:**
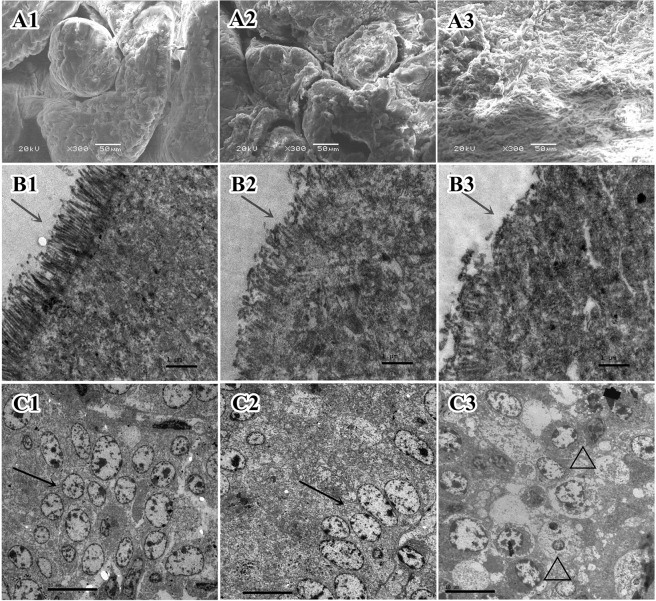
Electron microscopic micrographs of the duodenum in 42-day old piglets. (**A1**) SEM of the duodrenal mucosa of a Duan-Nai-An-treated piglet showing strong and thick villi. (**A2**) SEM of the duodenal mucosa of a yeast-treated piglet showing strong and thick villi and mucus residues. (**A3**) SEM showing the duodenal mucosa of a control piglet with villi sloughed off. (**B1**) TEM of the duodenal villi of a Duan-Nai-An-treated piglet. The microvilli were structurally intact and densely oriented (indicated by an arrow). (**B2**) TEM of the duodenal villi of a yeast-treated piglet. Microvilli were partially fractured (indicated by an arrow). (**B3**) TEM of the duodenal villi of a control piglet. Microvilli sloughed off (indicated by an arrow). (**C1**) TEM showing a large number of lymphocytes (indicated by an arrow) in the duodenal lamina propria of a Duan-Nai-An-treated piglet. (**C2**) TEM showing a large number of lymphocytes (indicated by an arrow) in the duodenal lamina propria of a yeast-treated piglet. (**C3**) TEM of the duodenal lamina propria of a control piglet showing necrosis, karyopyknosis and chromatin margination of lymphocytes (indicated by △).

The changes in the duodenum were more prominent. The duodenal mucosa of the Duan-Nai-An-treated group piglets were the most intact, with thick and healthy villi (Fig. 7A1). Partial villus loss were observed in the yeast-treated group (Fig. 7A2), and severe villus loss in the duodenum in the control group, resulting in exposure of lamina propria (Fig. 7A3).

Under TEM, the changes in duodenum were different among the groups at 42-days of age. The duodenum of the Duan-Nai-An treated piglets showed intact epithelial structure with dense and uniform microvilli that were perpendicular to the apical surface of mucosa (Fig. 7B1), and a large number of plasma cells and lymphocytes were present in the lamina propria (Fig. 7C1). However, the microvilli of yeast-treated piglet were partially fractured (Fig. 7B2) and there were a great number of lymphocytes in lamina propria of duodenum (Fig. 7C2). In the control piglets the duodenal striated borders varied in thickness with significant loss of microvilli (Fig. 7B3). Moreover, there were fewer plasma cells in the lamina propria and some of the cells showed nucleus vacuolization (Fig. 7C3).

#### Ileum

**Figure 8:**
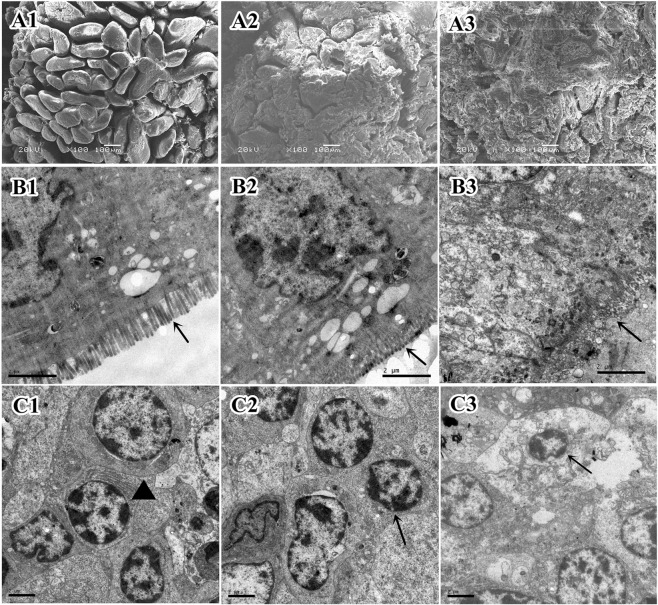
Electron micrographs of the ileum in 42-day old piglets. (**A1**) SEM of the ileal mucosa of a Duan-Nai-An-treated piglet showing intact and clearly visible villi. (**A2**) SEM of the ileal mucosa of a yeast-treated piglet showing localized mucus residues and partial slough of epithelium. (**A3**) SEM of the ileal mucosa of a control piglet showing a large number of adhering mucus, partial villi damaged, and slough of the epithelium. (**B1**) TEM of the ileal villi of a Duan-Nai-An -treated piglet showing clear, intact and dense microvilli (indicated by an arrow). (**B2**) TEM of the ileal villi of a yeast-treated piglet showing intact microvilli (indicated by an arrow). (**B3**) TEM of the ileal villi of a control piglet showing fractured and sloughed microvilli (indicated by an arrow)**. (C1**) TEM of the ileal lamina propria of a Duan-Nai-An-treated piglet showing well developed lymphocytes with abundant endoplasmic reticulum (indicated by ▲). (**C2**) TEM of the ileal lamina propria of a yeast-treated piglet showing well developed lymphocytes (indicated by an arrow). (**C3**) TEM of the ileal lamina propria of a control piglet showing necrosis and chromatin margination of lymphocytes (indicated by an arrow).

Under SEM, the ileal mucosa of the Duan-Nai-An group piglets were clear with thick, healthy and intact villi (Fig. 8A1). The ileum of the yeast-treated group also had mucus at the surface, but the villi were thick (Fig. 8A2). However, a large amount of mucus at the mucosal surface, thin villi with partial loss were observed in the control piglets (Fig. 8A3).

Under TEM, there were also differences in ileal structures among the groups at 42 days of age. The ileal epithelium of the Duan-Nai-An-treated piglets was intact with dense and uniform microvilli that were perpendicular to the apical surface of mucosa (Fig. 8B1). A large number of lymphocytes were present in the lamina propria and well-developed small lymphocytes were also visible (Fig. 8C1). The microvilli of yeast-treated piglets were neatly oriented (Fig. 8B2) and lymphocytes in lamina propria were well developed (Fig. 8C2). In contrast, the control group showed severe loss of epithelial microvilli (Fig. 8B3). Although many lymphocytes were seen in the lamina propria, many of them showed lesions such as mitochondrial swelling, nuclear condensation, and chromatin margination (Fig. 8C3).

## Discussion

The gastrointestinal mucosa constitutes the largest mucosal surface of the body. The intestinal surface area of adults is about 260–300 m^2^, equivalent to 200 times the surface area of skin^24^. Intestinal mucosa, where the body comes in contact with viruses, bacteria, food proteins, and other metabolites, is the first line of defense against infections from pathogenic microorganisms^1,2^. The barrier functions of intestinal mucosa include the mechanical barrier, biological barrier, chemical barrier, and immunological barrier^25^, among which the mechanical and immunological barriers play an important role. The integrity of intestinal mucosal epithelia (including columnar cells, goblet cells and entero-endocrine cells) is closely related to the mechanical barrier function that protects against pathogen invasion and infection. Additionally, the thickness of the intestine wall is also related to the physical barrier function and peristalsis. When the intestine wall becomes thin, peristalsis slows down and accumulation of undigested food causes abnormal fermentation, resulting in diarrhea. Moreover, reduced peristalsis results in retention of pathogens in the intestine and increases the chance of infection. Therefore normal intestinal peristalsis is important in food digestion and absorption, as well as in removing pathogens in a timely manner through bowel movement^26^.

Weaning is a big challenge for piglets. It is the key period of piglets’ life, and also the time to establish their future growth and development. The incidence of diarrhea caused by weaning stress has reached 30–50% in different region in China. Early-weaned stress causes a major damage to the intestinal tract^27^. The impact on small intestinal mucosa is particularly profound, altering the morphology, structure and function of the intestinal epithelia. Weaning induces both acute and long-lasting structural and functional changes in the small intestine including shortening of the villi (villous atrophy) and an increase in crypt depth^3,16^. For piglets, the small intestine is the main organ for nutrient absorption and transportation, and a healthy small intestinal mucosa is important for ensuring a normal digestion function as nutrient absorption relies heavily on the villi of small intestine. Intestinal villi are finger-like projections of the mucosal epithelial lining toward the intestinal lumen and are composed of enterocytes, goblet cells, and enterendocrine cells, whose functions are responsible for absorption and secretion^28^. As an important part of the small intestine, villi can repel harmful bacteria by strong and rhythmic movement.

Antibiotics are commonly used for managing weaning diarrhea. However, more and more livestock producers are seeking for natural alternatives to antibiotics and feed supplements to enhance animal health and growth performance. Probiotics, as a very hot topic in recent years, deliver clinical benefits to host health resulting from the combined action of diverse mechanisms. In some cases, the mechanisms are driven directly by interactions with the resident microbiota, such as the production of antimicrobial products and cross-feeding other resident microorganisms. In other cases, their effects might be direct via interaction with host immune cells^29^.

Yeast and its derivatives have long been proposed as prebiotic or probiotic alternatives in swine feed. There are many reports about the impact of yeast both directly and indirectly on the immune system and on enteric microbiota, thereby alleviating the negative effects associated with stress and disease^30^. For example, Zhang *et al*. report that yeast culture enhanced the immunity and disease resistance of gibel carp partly through TLR2 pathway^31^. Nochta, I. *et al*. reported that mannan-oligosaccharides derived from yeast fermentation products had positive effects on the immune system in weaned piglets^32^. Zhu.C *et al*. reported that live yeast and superfine yeast powders might enhance the intestinal immunity by regulating the secretion of mucosal SIgA and reduction of pathogen colonization, thus improving intestinal health of weaned piglets^33^. Che, L. Q. *et al*. reported live yeast supplementation in diet could alleviate the severity of diarrhea in piglets with enterotoxigenic *Escherichia coli* K88, which may be associated with the improved permeability, innate immunity and bacterial profile^34^.

Our previous study showed that the dietary supplementation with yeast cultures optimized intestinal flora, improved feed conversion ratio and health of the early-weaned piglets, and effectively helped piglets overcome the stress induced by weaning^23^. In this study, we provioded new evidence showing that Duan-Nai-An improved intestinal mucosal integrity in weaning piglets and the effect was particularly pronounced with the duodenal epithelia, where villi were wider and longer compared to the control group, and microvilli were intact and healthy with well-formed striated border (Fig. 7B1). This finding further explains how this yeast probiotic is beneficial for piglet health.

The intestinal morphology, especially the length and width of villi and the depth of crypt, is one of the main indicators for intestinal health of pigs. The margin of villi secretes a variety of digestive enzymes, therefore, the longer the villi, the stronger the digestible and absorptive capacity of the intestine. The depth of the crypt reflects the speed of mitosis of intestinal villi to produce epithelial cells. When the crypt becomes shallower, it indicates that the cell maturation rate and secretion function are increased. As a result, the V/C ratio can reflect the digestive and absorptive function of the small intestine. When the V/C ratio decreases, the digestive and absorptive capacity is decreased and often accompanied by diarrhea and growth retardation. On the contrary, the number of intestinal epithelial cells increased, the absorption area increased, and the absorption and utilization rate of nutrients increased^35^. Barrier loss is associated with disease risk. The lesion of intestinal barrier function has been reported as an early event in various intestinal and systemic disorders’ pathogenesis. It may result in increased permeability of the substance in the intestinal track, such as bacteria and their products (e.g. lipopolysaccharides), into the mucosal layer and may lead to local and systemic inflammatory responses. A large body of circumstantial evidence indicates that intestinal barrier dysfunction is associated with the pathogenesis of Crohn’s disease^36–38^. Based on the morphological and structural observations, it can be concluded that Duan-Nai-An helps to maintain the integrity of the intestinal mucosa, improves intestinal mucosa-associated immune function and gut health in weaning piglets.

It has been reported that weaning piglets supplemented with yeast proteins showed increased villous height in duodenum^39^. Muthusamy N. *et al*. reported that supplementation of whole Saccharomyces cerevisiae yeast increased villus height in the jejunum, width of villi in the ileum and number of goblet cells in villi of the jejunum and ileum in broilers^40^. Bontempo V. *et al*. reported that live yeast supplementation had positive effect upon intestinal morpho-functional aspects and is able to promote a “healthy” intestine in weaning piglets^41^. Martinez-Puig *et al*. found a yeast extract preparation increased intestinal villus height when fed to weaning piglets^42^. Results from our study are consistent with findings in the above reports.

The basal diet (Table 2) is the standard commercial diet for weaning piglets. Despite the fact that it provides sufficient nutrients, some piglets developed clinical diarrhea and this was especially obvious with the control group (Fig. 1B). The clinical diarrhea was likely due to the stress and other risk factors associated with the weaning process, which results in gut dysfunction and proliferation of enterotoxigenic *E. coli* in the intestine. It should be pointed out that this study was conducted on a commercial farm under normal production conditions. Thus the piglets were exposed to the risk factors of meaning and consequently some of them developed clinical diarrhea, Notably, supplement of the basal feed with Duan-Nai-An significantly reduced the diarrhea rate and death rate (Fig. 1B), indicating its efficacy in improving gut health.

**Table 2:** Nutrient compositions and values of the basal diet.

Basal diet composition	Content %	Nutrient Values	Content
corn	61.00	digestible energy* (MJ/kg)	13.86
soybean meal	12.56	crude protein content %	20.18
extruded full fat soybean	10.00	calcium%	0.96
middlings	5.00	available phosphorus%	0.63
fish meal	3.00	lysine* content %	1.54
whey powder	3.20	methionine and cysteine*	0.59
calcium phosphate, dibasic	1.40		
sodium chloride	0.34		
acidifier	1.00		
vegetable oil	1.50		
Premix^a^	1.00		

^a^Premix contains trace elements and vitamins. Every kg of basal diet contains 180 mg of iron, 100 mg of copper, 60 mg of magnesium, 180 mg of zinc, 12,000 U of vitamin A, 65 mg of vitamin E, 500 IU of vitamin D3, 0.5 mg of vitamin K, 4 mg of vitamin B1, 8 mg of vitamin B2, 3 mg of vitamin B6, 0.03 mg of vitamin B12, 30 mg of niacin, 15 mg of pantothenic acid, 0.7 mg of folate, and 0.4 mg of biotin. *Asterisk indicates the value was based on calculation, while the other nutrient values were obtained from experimental analyses.

The intestinal immunological barrier is primarily composed of the gut-associated lymphoid tissues. There are organized lymphoid tissue and scattered lymphocytes throughout the entire intestinal wall. The former includes aggregated lymphoid nodules or Peyer patches (PPs), solitary lymphoid nodules, and mesenteric lymph nodes, while the latter mainly refers to the scattered lymphocytes found in lamina propria and the epithelia. The intestinal lymphoid tissues play an important role in mucosal immunity^26^.

In 42-day-old piglets of Group I, there were many PPs scattered on the surface of jejunal, ileal, and colonic mucosa, especially scrotiform PPs in colonic mucosa. Under the microscope, there was no developed lymphoid tissue on the outer surface mucosa of the scrotiform PPs, with only scattered lymphocytes in the lamina propria. In contrast, there were developed lymphoid tissues in the entire inner wall. On the lateral surfaces, the lamina propria was almost filled with a large number of scattered lymphocytes; on the basal surfaces, there were well-developed lymph nodes near the circular muscle, which were composed of multiple lymph follicles, and the germinal center of each lymph follicle was clearly visible (Fig. 5). It was reported that Peyer’s patches (PPs) are the major organized lymphoid structures involved in the induction of mucosal immune responses in the intestine. It has been suggested that PPs are a site of B cell differentiation and a source of precursor cells for IgA production^43^. The PPs constituted the most important inductive site in the mucosal immunity of the gastrointestinal tract, and were mainly responsible for the ingestion, processing and presentation of antigens on the mucosal surface^44^.

It has been reported that stress induces apoptosis of intestinal lymphocytes in mice^45^, while probiotics increase the number of lymphocytes in mouse intestinal mucosa and reduce apoptosis^46^. As reported previously, an increase in the number of lymphocytes in the intestine represents enhanced intestinal mucosal immunity^47^. Bontempo*et al*. reported that when live yeasts were supplemented to the diet of weaning piglets, the number of lymphocytes and the height of villi increased in ileal epithelia^41^. In this study, it was observed that there were much more lymphatic tissues in the Duan-Nai-An-treated piglets (Group I) than in piglets of groups II and III as evidenced by I) proliferation of plasma cells in duodenal lamina propria; II) appearance of PPs and proliferation of lymphocytes with some entering into lamina propria in jejunal submucosa; III) well-developed lymphoid nodules in ileal submucosa, proliferation of lymphocytes into lamina propria and even into the epithelium lining, thick and strong villi, and distinct PPs in ileum; and IV)well-developed lymphoid nodules in mesenteric lymph nodes with clearly visible germinal centers containing distinct light and dark zones as well as spread of lymphoid nodules to near the capsule of lymph nodes (Fig. 6A). In contrast, the piglets from the control group showed atrophy of PPs in ileal submucosa, decreased numbers of lymphocytes accompanied by lymphocyte degeneration and necrosis in lamina propria, and poorly developed lymphoid nodules in mesenteric lymph nodes (Fig. 6C). The differences between groups 1 and III suggest Duan-Nai-An was able to maintain the normal immunological barrier function of the intestinal mucosa and improve mucosal immunity by promoting lymphocyte proliferation in lamina propria and lymphoid nodule development in mesenteric lymph nodes. It was also observed that Duan-Nai-An increased the number of plasma cells in intestinal lamina propria and promoted the proliferation of plasma cells in mesenteric lymph nodes.

How Duan-Nai-An improves intestinal integrity and immune function remains unknown, but one possible mechanism is through its beneficial impact on the gut microbiome. Previously we demonstrated that Duan-Nai-An was not only effective in improving the health and growth of early-weaned piglets but also significantly shaped the bacterial community of gut microbiome. Furthermore, 13 bacterial genera, including *Enterococcus, Succinivibrio, Ruminococcus, Sharpea, Desulfovibrio, RFN20, Sphaerochaeta, Peptococcus, Anaeroplasma*, and 4 undefined genera, were more abundant in Duan-Nai-An-fed piglets than in the control pigs, suggest that Duan-Nai-An helps to maintain a stable bacterial community in gut microbiota for early-weaned piglets^23^.

Among the 13 taxa, *Enterococcus* strains can produce bacteriocin^48^ and could also prevent the colonization and stabilization of pathogens unfriendly to the host by competing for adhesion sites^49^. *Succinivibrio* and *Ruminococci* play an important role in digesting the complex carbohydrates for the high cellulose degradation capacity^50^. *Saccharomyces* and *Enterococcus* are the gamma-aminobutyric acid (GABA) producing microorganisms^51^, GABA showed regulatory roles in the intestinal health and immunity^52^*. Sharpea* and *Desulfovibrio* are involved in the production of butyrate and other short-chain fatty acid (SCFA)^53^. SCFA are the final products of fermentation of dietary fibers by intestinal microbiota, and have multiple positive effects on animal energy metabolism^53,54^. The microbiota shaped by Duan-Nai-An may have contributed to the inhibition of pathogen colonization, improved the energy conversion of diet, and enhanced intestinal immunity, explaining the beneficial effect of Duan-Nai-An on improving performance and health in weaned piglets.

In conclusion, Duan-Nai-An as a diet supplement helps to maintain and improve the morphology and structure of mucosal epithelial cells as well as the integrity of the intestinal mucosa in the small intestine of weaning piglets. Duan-Nai-An also promoted the growth and development of lymphatic tissues in the intestine. Thus, Duan-Nai-An enhances the mechanical and immunological barrier functions of the intestinal mucosa and improves mucosal immunity of the small intestine. This beneficial effect may be related to its ability to optimize gut microbiome in pigs^23^. Future studies are warranted to further understand how Duan-Nai-An improves gut health and how it can be optimized for practical use in enhancing swine health.

## Materials and Methods

### Ethics

The experimental procedures performed in this study were approved by Animal Care and Use Committee of Lanzhou Institute of Husbandry and Pharmaceutical Sciences, Chinese Academy of Agricultural Sciences. It is strict accordance with the Regulations on the Management of Experimental Animals in China. All surgery was performed under sodium pentobarbital anesthesia and exsanguinated to death. All efforts were made to minimize suffering.

### Yeast probiotic preparation

Duan-Nai-An was provided by the Key Laboratory of New Animal Drug Project, Lanzhou Institute of Husbandry and Pharmaceutical Sciences, Chinese Academy of Agricultural Science. *Saccharomyces cerevisiae* S288c was used in this study to prepare the yeast probiotic (Duan-Nai-An) because it is tolerant to bile salt and low pH^55^. *Saccharomyces cerevisiae* fermented malt was prepared at 28 °C for 72 hours. The original *Saccharomyces cerevisiae* S288c strain grew poorly under the presence of egg white powder. It was tamed by supplementing malt broth with 1% egg white powder at the beginning, and gradually up to 8%. After tamed for one year, S288c grew well in malt broth with 8% egg white powder. Viable yeast cells in both *Saccharomyces cerevisiae* S288c fermented malt(Yeast) and *Saccharomyces cerevisiae* fermented malt with 8% egg white (Duan-Nai-An) were about 2.0 × 10^8^ CFU/ml. The survivability test showed that the viable CFUs in both *Saccharomyces cerevisiae* preparations did not change significantly after stored at 4 °C for 30 days^23^.

### Animals and experiment design

The study was performed on a commercial Swine Farm in Gansu Province under natural conditions. In total, 108 healthy 20 day-old piglets from eighteen litters (6 piglets per litter) were chosen for the study (Duroc × Yorkshire × Landrace crossbred pigs). During the test period, piglets were managed under normal production practices, food and water were given *ad libitum*. No experimental challenges with pathogens were given to the pigs, but some animals developed clinical diarrhea due to weaning stress and natural infection. Piglets were monitored once per hour and no treatment was administered when diarrhea occurred.

Eighteen litters of piglets were randomly divided into 3 treatment groups, with 6 litters in each group, which means in each group, there were 6 replicate subgroups, each of which consisted of 6 piglets (from the same litter). Group I was given the basal feed supplemented with the tamed *Saccharomyces cerevisiae* fermented egg white (Duan-Nai-An group), Group II was given the basal feed and the original *Saccharomyces cerevisiae* fermented malt (Yeast group). Group III was given the basal feed only (with no supplements) and served as a control group. The basal feed was a standard commercial diet used for piglets and its nutrient compositions are shown in Table 2. The experiment started when the piglets were 20-days old (before weaning). Piglets were observed for 3 days before given the supplements to ensure they were clinically normal. The piglets were fed with Duan-Nai-An (group I) or yeast (group II) for 10 ml/day at the age of 23 days by oral gavage and continued for 10 days. All piglets were weaned at 28-day old. Health evaluation (i.e. diarrhea rate, severe diarrhea rate, and death rate) were carried out as described previously^56^. At 23, 28, 35, 42-day of age, 6 pigs from each group (one from each subgroup or litter) were euthanized for examination. After the piglets were euthanized, the junction of each intestinal tract were ligatured; a 5 cm segment in the middle section of the duodenum, jejunum, ileum, cecum, and colon as well as mesenteric lymph nodes were collected from each piglet and fixed in 10% formalin at room temperature (for light microscopy) and glutaraldehyde solution at 4 °C (for electron microscopy). In total, 72 piglets were euthanized and samples were collected.

### Equipment and reagents

Equipment used in this study included paraffin microtome (HistoStat 820), Olympus BX-UCB/BX 61 light microscope and its image acquisition system, semithin microtome (JEOL JUM-7; Japan), ultrathin microtome (LKB V; Sweden), ion sputter (JEOL JFC-1600; Japan), scanning electron microscope (JEOL JSM-6380LV; Japan), and transmission electron microscope (JEM-1230; Japan). Reagents including Hematoxylin, eosin, differentiating solution (to remove excess hematoxylin dye), affixation agent, and 2.5% glutaraldehyde were purchased from Beijing Solarbio Science & Technology Co., Ltd.

### Sample preparation for light microscopy

Samples were fixed in 10% neutral formalin liquid at room temperature for 12 hrs, and the fixative was replaced and further fixed for 48 hrs. After that, samples were dehydrated in alcohol gradients (80%, 95%, and 100%), cleared with xylene, and embedded in paraffin wax. Paraffin-embedded samples were sectioned into 6 μm-thick slices and stained with Hematoxylin-Eosin (H&E). H&E-stained slides were examined under a light microscope and images were captured using an image collection system. The stained sections were evaluated for the structure and distribution of lymphoid tissue in the lamina propria and submucosa under the microscope. Meanwhile, the length and the width of the villi, as well as the depth of crypt were measured microscopically per 10 mm^2^ surface area (Image-Pro Plus 6.0). Each set of data included 6 samples which contained 5 consecutive slices. Every slice was measured in a fixed visual field.

### Sample preparation for electron microscopy

#### Scanning electron microscopy (SEM)

After piglets were euthanized, tissue samples of 0.5 × 0.5 × 0.2 cm^3^ in size were quickly collected from duodenum and ileum and fixed in 2.5% glutaraldehyde in phosphate buffer (0.1 M, pH 7.3) at 4 °C for at least 2hrs. Then the tissue blocks were washed twice (10 min each) in phosphate buffer (0.1 M, pH 7.2–7.4), and were further fixed in 1% osmium tetroxide for 1–2 hrs at 4 °C. After washing with phosphate buffer twice (10 min each), the tissue blocks were dehydrated in gradient alcohol and acetone (70% ethanol → 80% ethanol → 90% ethanol → 100% ethanol → 100% acetone → 100% acetone; 10 min each). The fixed and dehydrated tissue blocks were freeze-dried, coated with gold using an ion sputter (JEOL JFC-1600; Japan), and observed under a scanning electron microscope.

#### Transmission electron microscopy (TEM)

After piglets were euthanized, tissue blocks of ~1 mm^3^ in size were quickly cut from duodenum and ileum and fixed in a 2.5% glutaraldehyde solution for at least 2 hrs at 4 °C. Then the tissue blocks were rinsed twice in phosphate buffer (0.1 M, pH 7.2–7.4) for 10 min each time and fixed in 1% osmium tetroxide for 1–2 hrs at 4 °C. After rinsing in phosphate buffer twice (10 min each) and dehydration in ascending grades of ethanol, the specimens were embedded in Epon 812 according to standard protocols. Semithin sections (1 µm) were cut for preliminary localization and then ultrathin sections (60–90 nm) were double stained with uranyl acetate and lead citrate. The sections were examined and imaged under a transmission electron microscope.

### Data and statistical analysis

Incidence of diarrhea, Height and Width of villi, Depth of crypt and Height of villi to Depth of crypt (V/C) ratios were analyzed between groups. Statistical analysis was performed by one-way ANOVA using IBM SPSS Statistics version 22.0. Two-sided p-value of < 0.05 was considered statistically significant.

## Supplementary information

Supplementary Information.

Supplementary Information2.
