# Supplementary figures and images for: Duan-Nai-An, A Yeast Probiotic, Improves Intestinal Mucosa Integrity and Immune Function in Weaned Piglets
Source: Sci Rep. 2020 Mar 12;10:4556. doi: 10.1038/s41598-020-61279-6 (PMC7067797; doi:10.1038/s41598-020-61279-6)

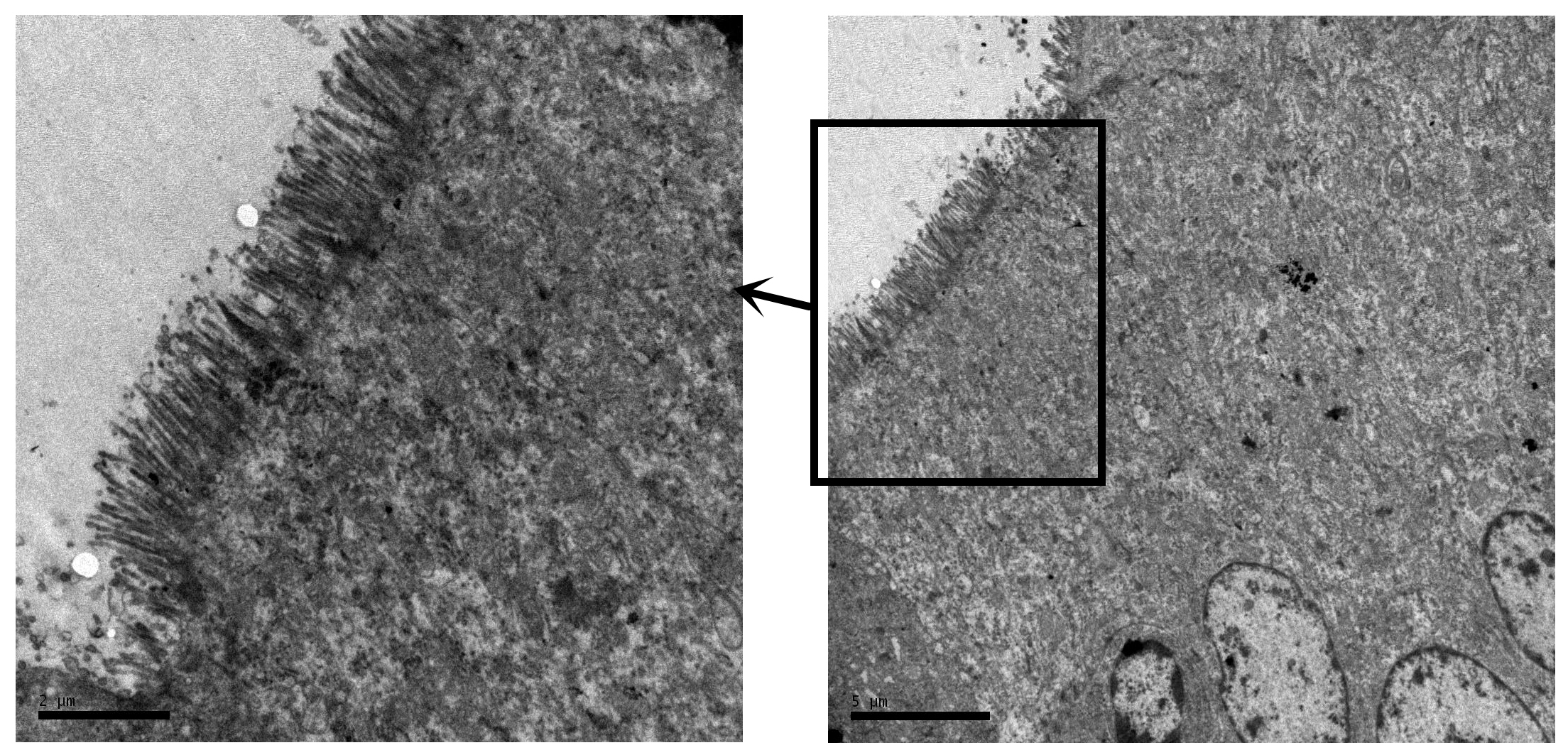

Supplement: Supplementary file 1 — Supplementary Information. [file 41598_2020_61279_MOESM1_ESM.tif]

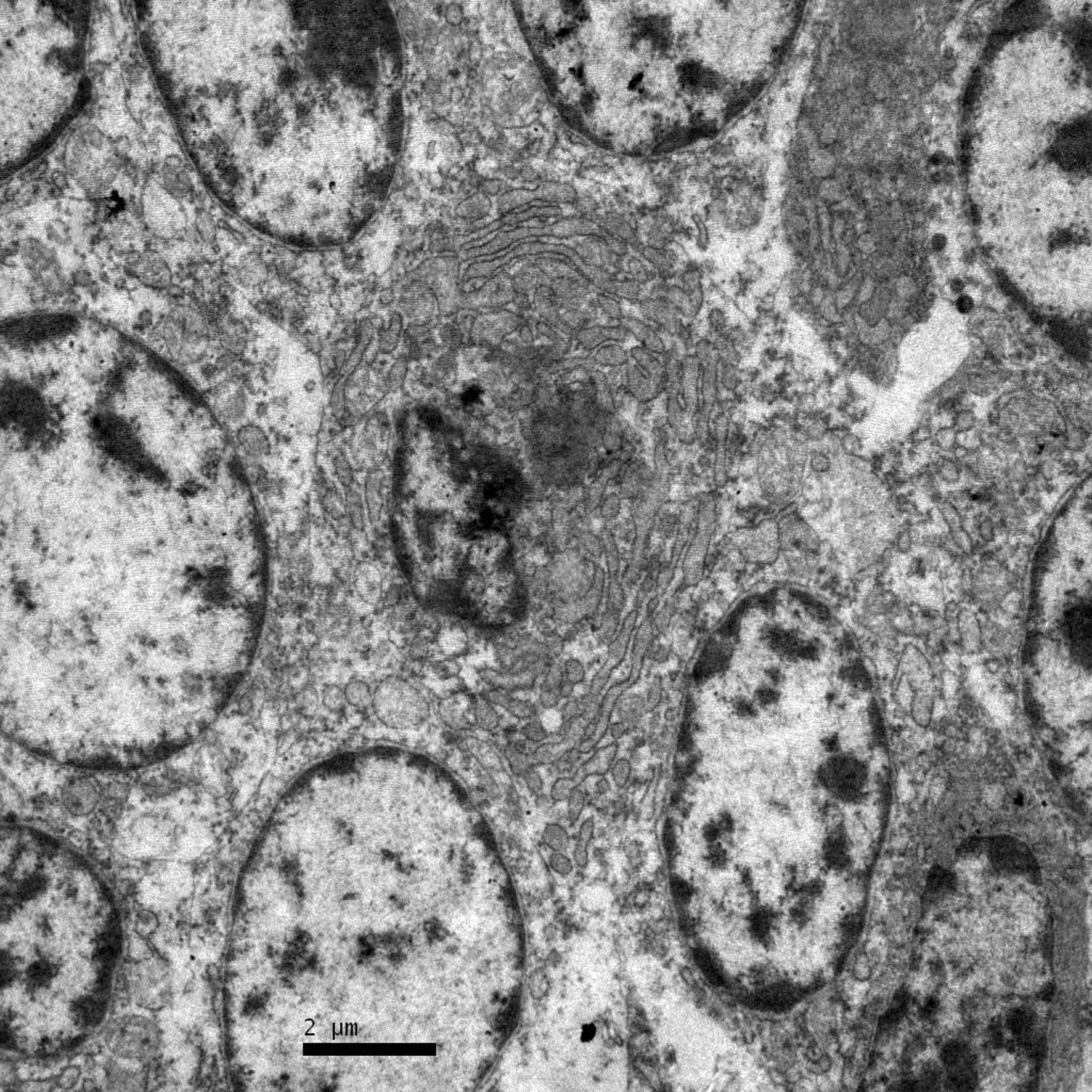

Supplement: Supplementary file 2 — Supplementary Information2. [file 41598_2020_61279_MOESM2_ESM.tif]
